# Ball-Milling Processing of Hydrogenated NdFeB Powders from the Recycling of End-of-Life Magnets

**DOI:** 10.3390/ma19173797

**Published:** 2026-09-06

**Authors:** Amanuel Elias Wako, Pablo Rodríguez, Beatrice Muzzi, Giovanna Trevisi, Laura Grau, Martin Albino, Tomaž Tomše, Benjamin Podmiljsak, Carlo Burkhardt, Franca Albertini, Claudio Sangregorio, César de Julián Fernández

**Affiliations:** 1 Department of Mathematical, Physical and Computer Sciences, University of Parma, Parco Area delle Scienze 7/A, 43124 Parma, Italy; amanuelelias.wako@unipr.it; 2 Institute of Materials for Electronics and Magnetism, CNR, Parco Area delle Scienze 37/A, 43124 Parma, Italy; giovanna.trevisi@imem.cnr.it (G.T.); franca.albertini@cnr.it (F.A.); 3 Independent Researcher; 4 Institute of Chemistry of OrganoMetallic Compounds, CNR, 50019 Sesto Fiorentino, Italy; beatrice.muzzi@cnr.it (B.M.); martin.albino@cnr.it (M.A.); csangregorio@iccom.cnr.it (C.S.); 5 Institute of Strategic Precious and Technology Metals, Pforzheim University, Tiefenbronner Str. 65, 75175 Pforzheim, Germany; laura.grau@hs-pforzheim.de (L.G.); carlo.burkhardt@hs-pforzheim.de (C.B.); 6 Department for Nanostructured Materials (K7), Jožef Stefan Institute, Jamova Cesta 39, 1000 Ljubljana, Slovenia; tomaz.tomse@ijs.si (T.T.); benjamin.podmiljsak@ijs.si (B.P.)

**Keywords:** NdFeB magnets, hydrogen decrepitation, ball milling, particle size reduction, magnet-to-magnet recycling process

## Abstract

Rare-earth-based permanent magnets are key elements for today’s technologies. Their recycling is crucial for securing the supply of critical raw materials and ensuring a sustainable circular economy in magnet production. In this study, hydrogen decrepitated NdFeB powders recovered from end-of-life magnets were processed using planetary ball milling to obtain powders suitable for recycled magnet production. The magnets were sourced from a wind turbine, scooter motor, and ring magnet, and exhibit different compositions and properties. The structural, morphological, and magnetic properties of the hydrogenated and ball-milled powders were investigated considering the effects of the milling time and ball diameters. Submicron-sized powders were achieved within 10 min with 5 mm balls, while longer milling times were required to obtain similar particle size reduction with 10 mm balls. Milling resulted in structural damage to the hydrogenated powders. Furthermore, although milling decreased the magnetization of the powders, an increase in the coercive field was observed for certain milling times. Micrometric powders sourced from the wind turbine, milled for 10 min using 5 mm balls, exhibited the highest coercive field of 0.27 T. This behavior is discussed considering the effect of the particle size reduction, the structural damage, the particle agglomeration, and the local compositional changes. We conclude that the powders produced from different source magnets exhibit similar patterns of morphological, structural, and magnetic changes under milling, and only the Tc and the Hc of the powders depend on their original magnet. Ball milling represents a promising technique for particle size refining in the hydrogen processing of magnetic scraps, a crucial step in the magnet-to-magnet recycling process.

## 1. Introduction

Rare-earth (RE) permanent magnets are key components in today’s technologies such as electric vehicles (EVs), wind turbines, consumer electronics, industrial motors, hard drives, robots, and sensors. NdFeB-type magnets offer excellent magnetic properties such as high maximum energy product, remanence, and coercivity [1,2]. Due to their essential role, especially in green energy technologies, the demand for NdFeB magnets continues to grow [3,4,5].

The long-term sustainable use of NdFeB magnets is challenged by supply chain vulnerabilities and geopolitical risks, notably due to China’s dominance in almost the entire magnet value chain [4,5]. This challenge drives the necessity to explore sustainable alternatives to ensure the supply of rare-earth elements (REEs) and RE-permanent magnets. In this regard, recycling NdFeB magnets from end-of-life (EoL) products is a promising approach for ensuring long-term REE sustainability and simultaneously promoting the circular economy [6]. Given the growing demand for NdFeB-type magnets, the development and implementation of efficient recycling methods have gained significant attention [7,8]. Substantial achievements have been made so far in terms of recovering recycled materials with competitive magnetic properties. At laboratory scale, the overall properties of the recycled NdFeB-sintered magnets have been demonstrated to be comparable to the original magnets [9,10,11].

EoL NdFeB magnets can be recycled using several methods, such as pyrometallurgy, hydrometallurgy, electrometallurgy, and direct magnet-to-magnet recycling [12,13]. The hydrogen processing of magnet scraps (HPMSs) is a highly promising magnet-to-magnet recycling method [14]. The HPMSs allows for obtaining hydrogenated NdFeB powders that can be further processed to produce new magnets [15,16]. Critical problems associated with magnet recycling, such as their demagnetization and the removal of certain corrosion-protection coatings, can be simply overcome with this process. Importantly, it offers a more efficient, cost-effective, and environmentally friendly approach for recycling NdFeB magnets as compared to hydrometallurgical and pyrometallurgical processes [13].

HPMSs is based on the strong hydrogen absorption by REEs and the subsequent decrepitation effect. Hydrogen is absorbed by the RE-rich grain boundary phase, resulting in the formation of RE hydrides, while it is also incorporated interstitially into the hard-magnetic Nd_2_Fe_14_B matrix phase. The difference in lattice expansion between these phases induces internal stresses, causing intergranular and transgranular cracks and phase segregation, and breaks down the magnet into friable coarse powders. This process is termed hydrogen decrepitation (HD) [17]. The resulting powders exhibit weak remanent magnetization and a low coercive field, which makes them easier to handle. After hydrogenation, the powders are further processed to refine the particle size while maintaining control over particle morphology to obtain micrometer-sized particles suitable for optimal magnetic performance in the final magnets. In the later processing stage, degassing is performed to induce the formation of the hard 2:14:1 phase. These powders can be used to produce bonded magnets or aligned in an external magnetic field and compacted for sintering to full density [18,19].

Understanding the intermediate steps in this process is under investigation. Different studies pay attention to the critical role of oxygen in the HPMSs powders, the shape of the particles [20], and the presence of possible coating-related contaminants and corrosion [21] in the final magnets. Other studies report further modification of the recycling process, e.g., introducing leaching procedures to eliminate oxides and RE compounds [22] or including RE-H in the magnet production process [23].

Controlling the morphology and grain size of the HPMSs powder is a key factor for good magnet properties. The HD process produces powders with particle sizes in the range of tens of hundreds of microns [14]. Thus, further processing is required to reduce the particle size to the optimal value to achieve high coercivity and balance between oxygen content and powder alignment -. The jet milling technique is commonly employed for further particle size reduction after the HD process [24,25]. Alternatively, the high-energy ball-milling (BM) method can be applied to further reduce the particle size of the hydrogenated coarse powders [26]. This technique is suitable for laboratory-scale studies that can be scaled up to industrial production. However, high-energy ball milling is a complex process that requires careful optimization of milling parameters to ensure uniform particle size reduction.

An important aspect of recycling NdFeB magnets is the origin of the waste. In fact, scraps from different applications could have different chemical compositions, microstructural characteristics, coatings, oxidation levels, aging, etc. As previously discussed, all these parameters can influence the final properties of the recycled magnets. Magnet recycling processes have been widely researched in recent years; however, the properties of the hydrogenated powders and the effects on their processing and the final magnets remain largely unexplored. In fact, the HD process has been mainly investigated in hard magnetic phases with controlled stoichiometry, while the study of multi-element magnets with complex structure has not yet been systematically studied.

In this study, we investigate the BM process on hydrogen decrepitated powders obtained from EoL scrap Nd-Fe-B-based magnets. The changes in the structural, morphological, and magnetic properties of EoL material after the HD process and the further BM processing were investigated. The ball diameter and milling durations were varied to assess their impact on the particle size of powders and on the change in the magnetic properties. These studies were performed in three different EoL magnets with different compositions and properties. Our comparative study shows the varying evolution of the processed-material properties. Our results demonstrate that the relationships among the properties of the EoL magnets, HD powders, and processed powders are not straightforward.

## 2. Materials and Methods

In this study, three types of NdFeB powders obtained from different recycled scrap magnets were investigated. The EoL magnets were extracted from a wind turbine (labeled M1_68); a ring magnet (labeled RMI); and a motor of a scooter (labeled S21). The three magnets were pulverized by HD in a hydrogen reactor under hydrogen atmosphere of 3 bar at room temperature. The resulting coarse powders were then transferred to a glove box under argon atmosphere to avoid oxidation or contamination. The powders were processed using a Fritsch “Pulverisette 7” high-energy planetary ball mill that operates at a constant rotation speed of 225 rev/min, and using hardened steel jars and balls, which is placed permanently inside a glove box. The jars have a diameter and height of 50 mm. The milling was performed using balls of two sizes. First, 5 mm diameter balls were used for durations of 5 to 150 min. Secondly, 10 mm diameter balls were used for processing durations ranging from 5 to 210 min. To avoid overheating, a 5 min cooling interval was allowed every 15 min. A ball-to-powder weight ratio of 20:1 was employed to achieve a particle size to a micron size range considering that the previous work [26] reported larger particle sizes using a smaller ball-to-powder ratio. Labeling “_HD” indicates the hydrogenated powders while “_BM xm” indicates the powders were ball milled for x minutes. The samples were stored, weighted and manipulated for further characterization inside a glove box with an inert argon atmosphere.

The morphology of the powders was investigated by scanning electron microscopy (SEM) using a Auriga Compact field-emission SEM (Zeiss, Oberkochen, Germany), equipped with an Oxford Xplore 30 Energy Dispersive Spectroscopy (EDS) system for local composition analysis. Particle size histograms were obtained from higher-magnification SEM images using ImageJ 1.54g software (National Institutes of Health, Bethesda, MD, USA). The data were fitted with a lognormal distribution to calculate the D_50_ and the lognormal standard deviation. Powder X-ray diffraction (XRD) data were recorded using 2 diffractometers working in Bragg–Brentano configuration, recorded over a 2θ range of 20–70° (0.01°; step size): an XRDynamic 500 diffractometer (Anton Paar, Graz, Austria) equipped with a Co Kα radiation source (λ = 1.79026 Å) and a Miniflex 600 144 diffractometer (Rigaku Corp.,Tokyo, Japan) equipped with Cu Kα radiation (λ = 1.5406 Å) and electronic filtering of Fe fluorescence. The crystal phases were first identified with JCDS ICDD cards. Afterward, the structural data were obtained using the Rietveld refinement fitting method employing HighScore Plus software (v3.0.5) by PANanalytical (Almelo, The Netherlands) [27].

The composition of the powders was analyzed by inductively coupled plasma (ICP) analysis using iCAP 7400 ICP-OES from Thermo Fisher Scientific Inc. (Waltham, MA, USA). The magnetic properties were always measured at room temperature with a Vibrating Sample Magnetometer (VSM, model 750, Lakeshore, Westerville, OH, USA), with a maximum field of 1.7 T) and with a DynaCool PPMS(Quantum Design, San Diego, CA, USA) with a maximum field of 9 T. Thermomagnetic analysis (TMA) was performed using a home-made susceptometer that reaches temperatures up to 900 °C to measure Curie temperature and for phase identification. The study was developed by producing approximately 50 g of HD powders, which required processing several RMI and S21 magnets; 1 g of HD powders was milled to obtain a batch of milled powders. Approximately 20–30 mg was employed for each characterization. The experimental repeatability and measurement uncertainty are limited to the possible differences among the pieces of the EoL magnets in each family. The calculated errors in the lattice parameters were ±0.001 Å, while the errors in the specific magnetization, the coercive field and the Curie temperature were ±1 A·m^2^/kg, ±1 mT and ±2 °C, respectively.

## 3. Results and Discussion

### 3.1. EoL Magnets

The three classes of EoL magnets have specific properties concerning the different applications in which they were employed. Table 1 summarizes the elements analyzed by ICP that compose each magnet after the hydrogenation process, which is representative of the original magnet composition. Each magnet presents different elemental content including rare earths (Nd, Pr, Gd, Dy, Ho, Tb, Ce), transition metals (Fe, Al, Co, Cu, Nb) and metaloids (B, Si, P). Considering the total content of rare earths, M1_68 is richer (38%wt) than the other two (around 30%wt), and always above the stoichiometric ratio Nd2: Fe14: B1 of the hard-magnetic phase (26.7%wt). The neodymium content of the wind turbine magnet M1_68 is 33.58%wt, larger than that of the others (20–21%wt), but it also incorporates Pr (0.87%) and Dy (3.27%). Pr is also present in large amounts in the RMI and S21 (4–5%wt) magnets. Heavy rare earth elements, Dy, Tb, and Ho, are present in the ring magnet RMI. Magnets from the scooter, S21, contain other rare earths: Gd and Ce. Cobalt, a strategic raw material [28], is present in the M1_68 and RMI magnets.

**Figure 1:**
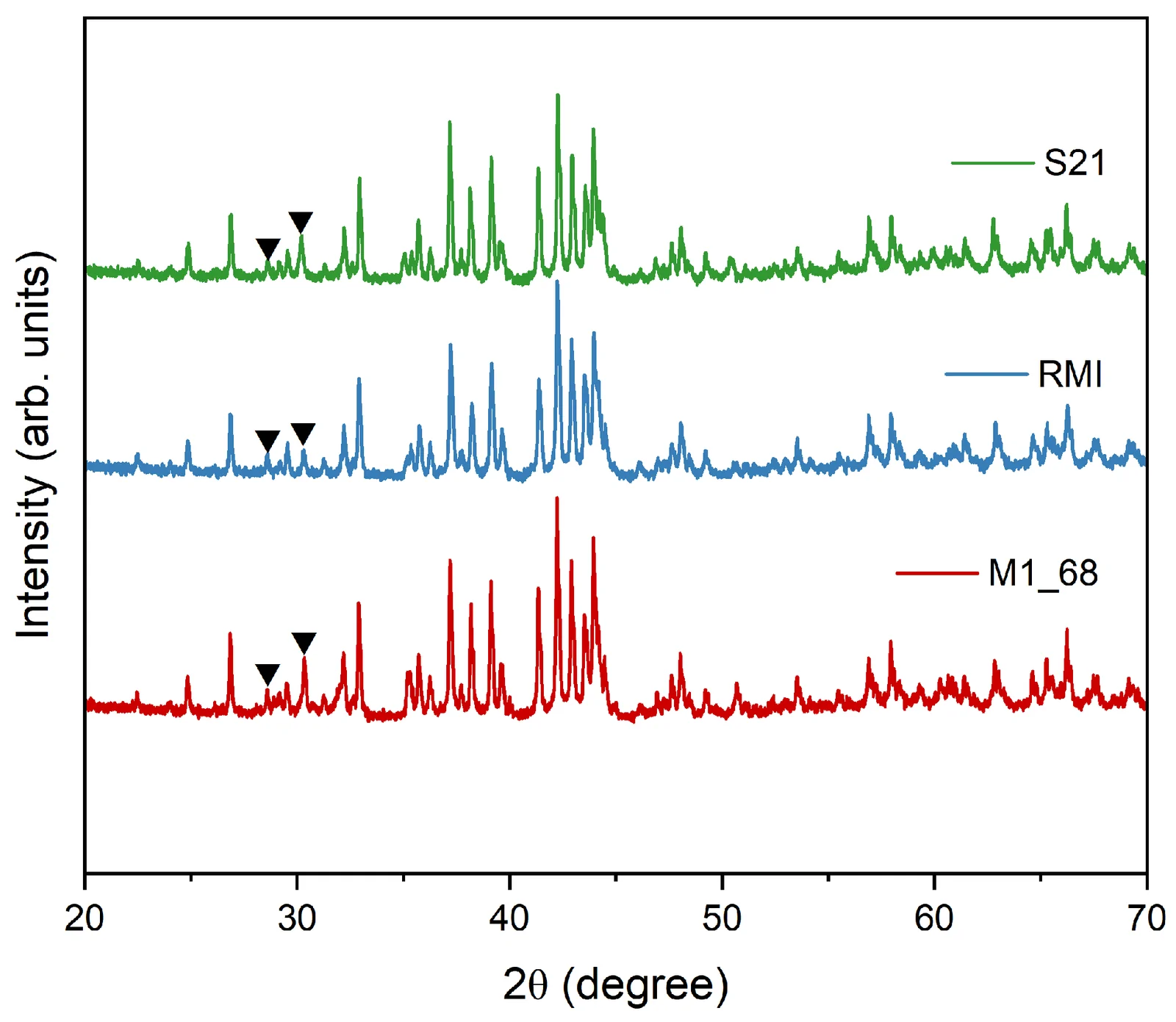
X-ray diffraction patterns of the powders from the EoL magnets. All unindexed peaks correspond to the space group P4_2_/mnm of NdFeB. ▼—corresponds to Nd_2_O_3_ phase.

The XRD patterns of powders from the three magnets are shown in Figure 1. All patterns correspond to the tetragonal Nd_2_Fe_14_B phase of space group P4_2_/mnm. The lattice parameters and cell volumes were calculated using the Rietveld refinement method. The results are summarized in Table 2. Figure S1a shows the experimental XRD patterns and the corresponding theoretical Rietveld profile of M1_68, as representative of the analysis of all the magnets. The weighted profile residual (Rwp) and the profile residual factors (Rp) from the Rietveld analysis are both below 10%, indicating a reliable fit to the lattice parameters. The calculated lattice parameters and cell volumes are summarized in Table 2. The cell parameters are similar between the three magnets and slightly smaller than those of Nd_2_Fe_14_B (a = 8.80 Å and c = 12.21 Å), with an estimated error of 0.001 nm) [1]. The differences between the intensities of some peaks of the experimental and fitted patterns were observed. This is an indication of the multi-element composition of the magnets. The crystal size of the powders was evaluated using the Scherrer equation. The width of peaks at half of the maximum intensity was similar to the instrumental width (3.5 mrad). This indicates that the crystal size should be over the micron scale, but it cannot be accurately determined; hence, it is not reported here. Finally, the peaks at 26.8°, 28.4° and 30.4° 2θ identify the presence of cubic Nd_2_O_3_ (JCPDS card n. 00-101-0281).

**Figure 2:**
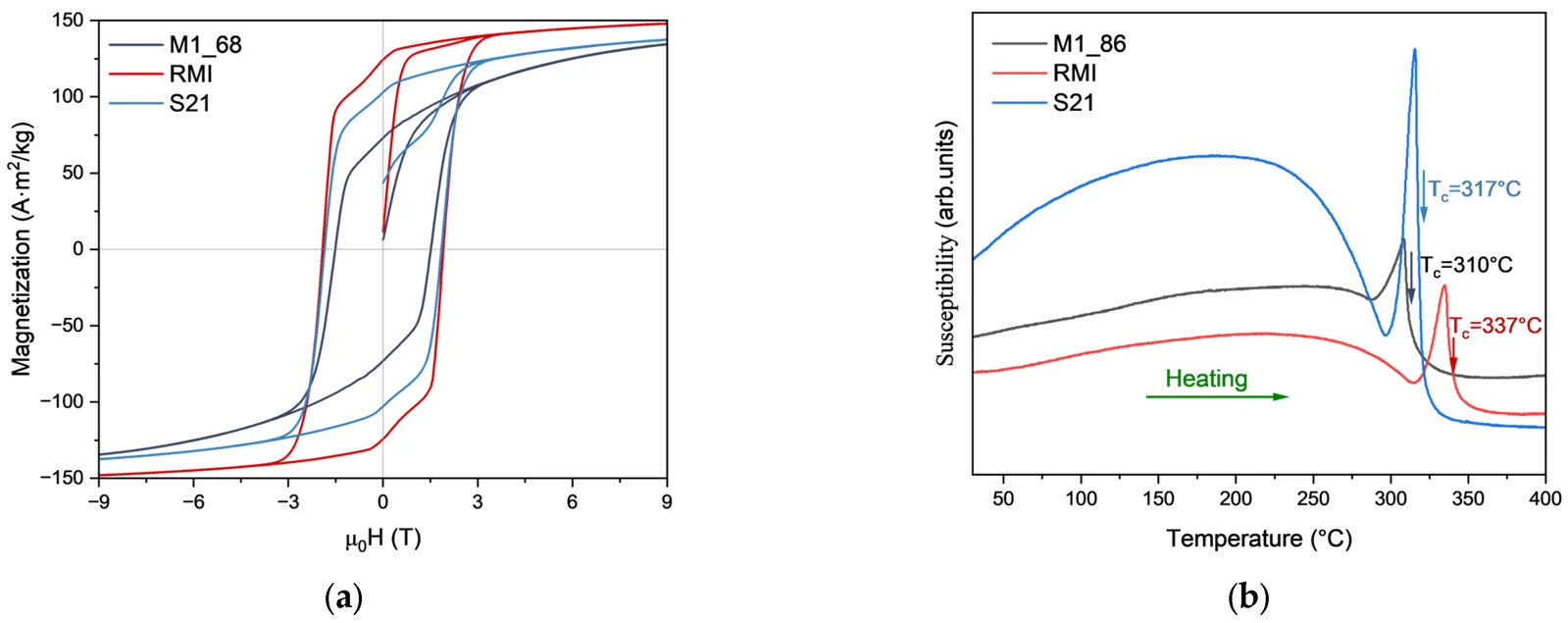
(**a**) Hysteresis loops and (**b**) thermomagnetic measurements of the EoL magnets.

The magnetic properties of the starting magnets are shown in Figure 2 and summarized in Table 3. All hysteresis loops were recorded from EoL pieces of the magnets when applying a maximum field of 9 T. The hysteresis of M1_68 and RMI exhibit some kinks near the remanence. This effect can be correlated to the irregular shape of the measured EOL pieces. In this case, regions of the magnets can have stronger demagnetization fields than the whole piece and reverse at smaller fields, giving rise to the kinks in the hysteresis. The specific magnetizations and coercive fields are in the range of 137–147 A·m^2^/kg and 1.8–1.9 T, respectively. The RMI magnet has the highest specific magnetization and coercive field (Hc), approximately 147 A·m^2^/kg and 1.9T, respectively.

The TMA curves representing the temperature dependence of the susceptibility (Figure 2b) exhibit the characteristic Hopkinson peak [29] produced at the Curie temperature (Tc) of the magnets. This temperature is evaluated as the maximum of the derivative in the drop of the susceptibility after the Hopkinson peak. Tc is a property correlated to exchange interactions in the magnetic structure at short and long distances. Hence, it depends mainly on the elemental composition of the materials. The M1_68 magnet has a Tc similar to that of the Nd (2:14:1) phase (312 °C), though it is composed of other REEs in which the corresponding 2:14:1 phase has a different Tc. In this case, the hard phase is composed of Nd and also by Pr and Dy, which the corresponding Tc values are smaller (292 °C) and larger (325 °C), respectively, than that of the Nd (2:14:1). The S21 magnet has a larger Tc (317 °C) than previously recorded. The increase in Tc can be due to the contribution from the Tc of the Gd 2:14:1 phase (385 °C), and can be dominant on the reduction due to Ho (300 °C) and Pr. In the RMI, in addition to the Pr, Ho and Dy elements, there is Tb with a higher Tc (347 °C) that gives rise to the EoL magnet with the highest Tc (337 °C) [30,31]. In addition, the Co content can increase the Curie temperature of the Nd (2:14:1) phase [32].

The differences in composition are related to the magnetic properties required for the specific applications, including different magnetization, remanence and coercive field values, working temperature conditions, and mechanical properties. In addition, they may also be dictated by economic and supply reasons.

### 3.2. Hydrogenated Powders

**Figure 3:**
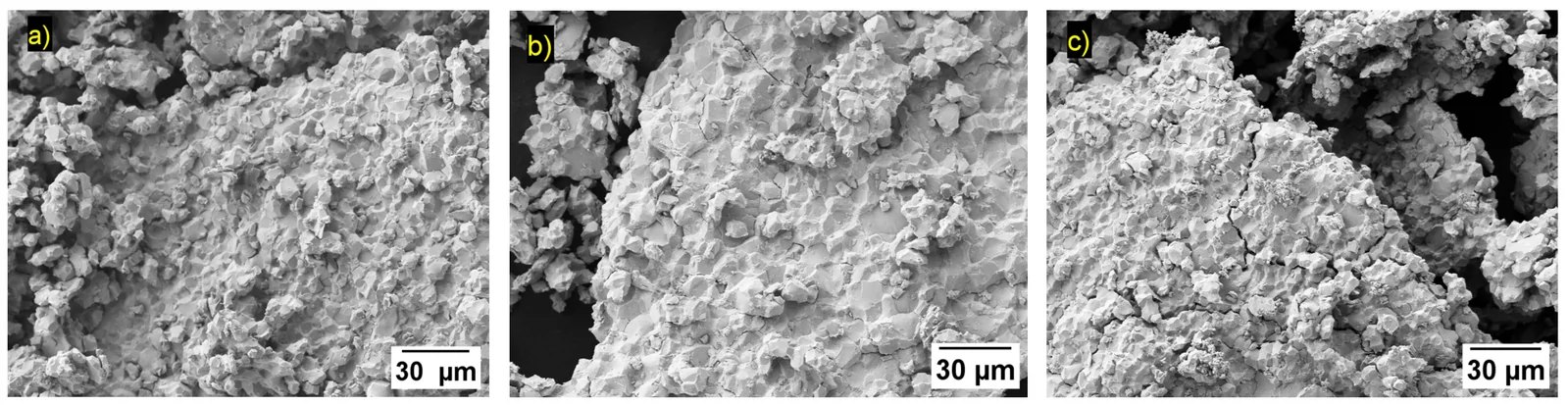
SEM images of hydride powders: (**a**) M1_68, (**b**) RMI, and (**c**) S21.

The EoL magnets were effectively broken down into coarse powders by a hydrogen decrepitation (HD) process. As shown in Figure 3a–c, the SEM studies reveal that the magnets are broken into submillimeter-sized powders. The powders consist of distinct polyhedral grains ranging from approximately 4 to 10 μm in size. All the EoL magnet powders reach several tens of microns across. The particle size histograms reported in Figure S2 show that the average size and lognormal standard deviation (Table 4) are similar in the M1_68 and S21 powders, with average sizes of 22 μm and approximately 40, respectively. The particles of the RMI sample are larger, 30 μm, and the distribution is broader, 46. Detailed studies also indicate the presence of intergranular cracking of grains and the absence of transgranular cracks. This morphology agrees with the general description of how hydrogen decrepitation performed at room temperature affects the intergranular regions [33].

**Table 4:** Average particle size, D_50_, and lognormal standard deviation of the particle size histograms of the HD powders, and those milled for 10 min using 5 mm balls and those milled for 150 min using 10 mm balls.

Sample	D_50_ (µm)	Std
M1_68_HD	21.6	38.6
RMI_HD	30.7	45.9
S21_HD	23.0	41.0
M1_68_BM10m	1.6	1.8
RMI_BM10m	1.7	3.4
S21_BM10m	1.5	1.0
M1_68_BM150m	3.0	4.5
RMI_BM150m	2.9	3.4
S21_BM150m	3.7	3.6

The possible elemental contamination of the powders due to the fragmentation of the steel balls and jars under milling was investigated using EDX. The Cr content, absent in the magnets but present in the stainless steel (17%wt), was evaluated in all milled samples. No Cr presence was detected, confirming the absence of elemental contamination.

**Figure 4:**
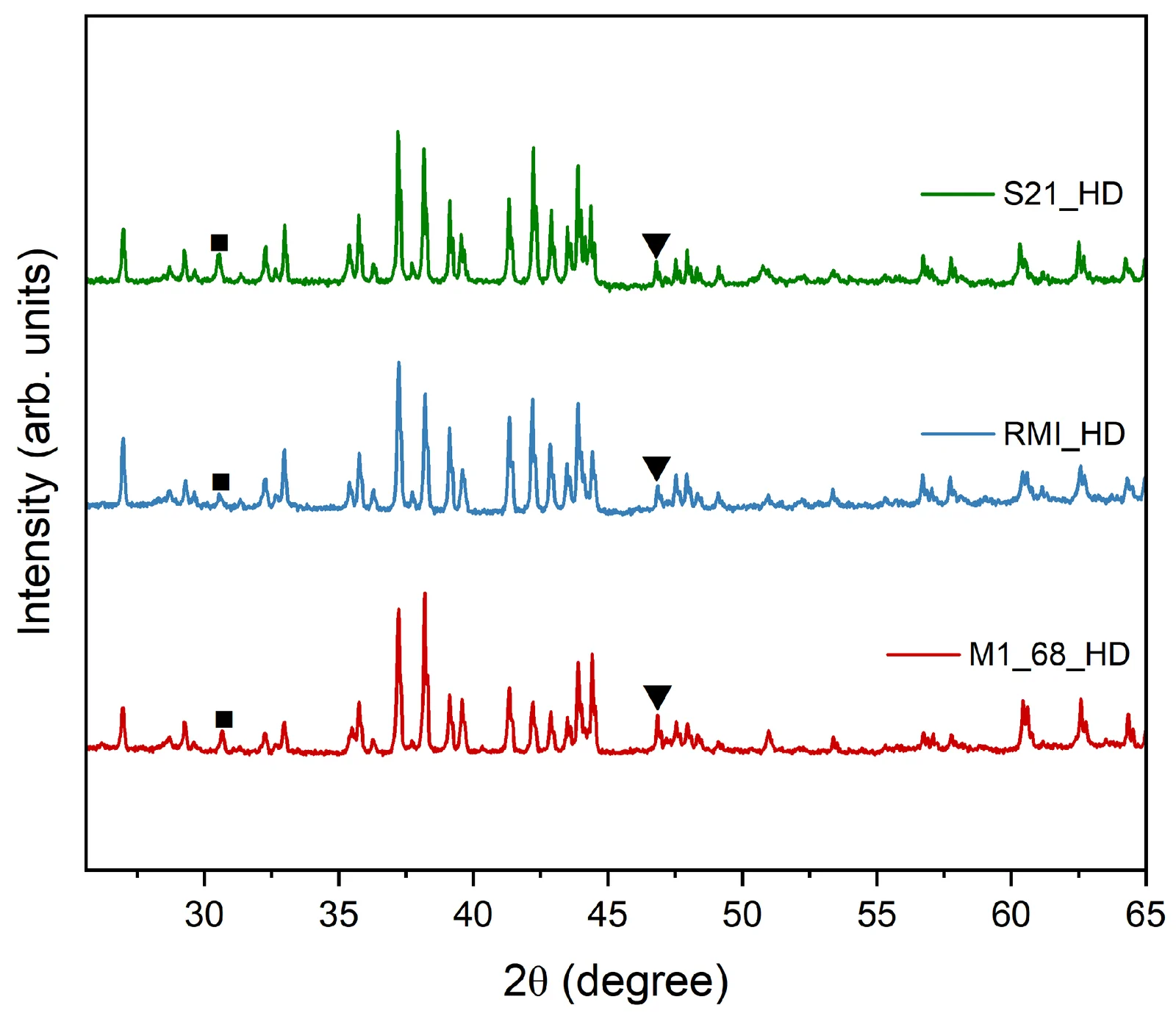
XRD patterns of the powders after hydrogen decrepitation. Symbols ▼ and ■ correspond to Pr_2_O_3_ and Nd_2_O_3_ phases, respectively.

The XRD patterns for all HD powders are presented in Figure 4. The observed peaks correspond to reflections of the tetragonal P4_2_/mnm symmetry, and the lattice parameters, calculated by the Rietveld refinement, are reported in Table 5. An example of the XRD pattern, together with the calculated Rietveld profile, is shown in Figure S1 for the hydrogenated powders from the RMI magnet. The Rwp and Rp from the Rietveld analysis are both below 5%, indicating a reliable fit. The differences in intensities between the experimental and fitted patterns provide evidence of the multi-element composition. The lattice parameters of the hydrogenated powders are larger than those of the EoL magnets. This indicates that lattice expansion induced by hydrogen absorption is the primary cause of magnet decrepitation. As with the EoL magnets, the widths of the peaks at half the maximum intensity were similar to the instrumental width; thus, the crystal size should be greater than a micron, but it cannot be accurately determined. No peaks corresponding to RE hydrides are observed within the technique’s limits. The diffraction spectra also reveal the presence of small amounts of rare earth oxides, in particular cubic Nd_2_O_3_ (JCPDS card n. 00-101-0281) and Pr_2_O_3_ (JCPDS card n. 00-101-0280).

The lattice expansion following hydrogen decrepitation is confirmed. The percentage of expansion in lattice and cell volume is summarized in Table 6. The lattice expansions are similar between parameters a and c, indicating that hydrogen occupied the interstitial sites [34]. The lattice expansions are similar for RMI and S21, while M1_68 shows the smallest change. This could suggest that the hydrogen content in RMI and S21 is higher than in the other magnet. On the other hand, the REE content in the M1_68 is the highest. These results indicate that the total hydrogenation of the magnetic phase depends on the REE content and composition of the EoL magnets.

**Figure 5:**
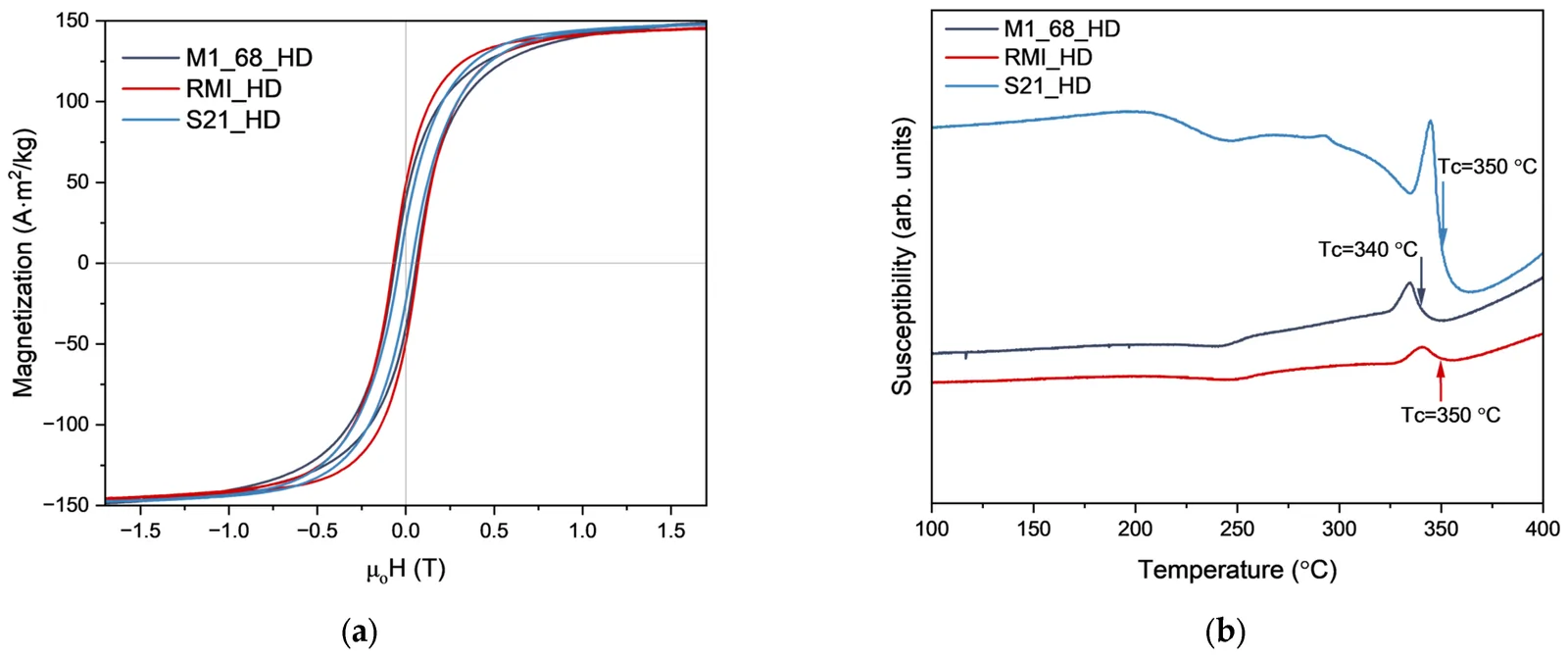
(**a**) Hysteresis loops and (**b**) thermomagnetic measurements of the HD powders.

Figure 5a presents the hysteresis loops measured at room temperature in the field interval up to 1.7 T, and Figure 5b presents the thermomagnetic analysis results. The saturation magnetizations of the three types of powders are similar (approximately 146 A·m^2^/kg); this is above the values measured in the original M1_68 and S21 EoL magnets. Also, they have small coercive fields, which vary between 30 mT (S21) and 70 mT (RMI). Finally, their Curie temperature is around 350 °C, a temperature that is larger than that of their respective EoL magnets.

In general, all the magnetic features of the HD powders appear uniform, as shown in Table 7. There are small differences that mainly concern the coercive field and the Tc. The coercive field of the respective hydrogenated powders decreases from RMI, M1_68 to S21, always in the range of tens of mT. The observed coercive field can be attributed, in the first instance, to the reduced magnetocrystalline anisotropy of the hydrogenated Nd-Fe-B grains [35]. In fact, the anisotropy field of hydrogenated Nd-Fe-B has been reported to be around 2–3 T [36], although it may vary depending on how much hydrogen is absorbed. In addition, the microstructural-related effects, such as defects, sharp edges or borders, may also give rise to the reversal processes, resulting in a decrease in coercivity [37,38,39,40].

In contrast, Tc increases in comparison to the values of the EoL magnets. The increase in Tc of the hydrides of the 2:14:1 with respect to the metallic pristine is well reported in the literature [41]. Both in Nd and in other REE 2:14:1 phases, Tc increases proportionally to the H content [42]. In this case, we observe that the increase in Tc is much larger in S21 and M1_68 than in RMI. On the other hand, we observe that the volume expansions due to the hydrogen interstitial content are similar in RMI and S21, larger than the values observed in M1_68. This coincides with the same Tc values for RMI and S21, which are larger than the M1-68 value. We conclude that the final Curie temperature of the hydrogenated powders can depend on their REE composition, as it occurs in the pristine magnets. In addition, the elemental composition determines the hydrogen absorption and hence the crystal features, the exchange interactions and, at a second level, the Curie temperature.

In addition, the thermomagnetic measurements (Figure 5b) of the HD powders exhibit a broad, shallow peak around 220 °C, mainly for S21. This indicates the presence of a magnetic secondary phase that is not observed in the XRD patterns. The presence of the secondary phase could be correlated to changes in the REE composition of the hydride phase with respect to the nominal one. These results bring forward a complex scenario that indicates how the original properties of the magnets are modified into the final properties of the hydrides. Consequently, the magnetic properties of the hydrides cannot be extrapolated from those of the corresponding pristine magnets.

### 3.3. Ball-Milling Processing

Processing using high-energy ball milling was performed on the HD powders to pursue their particle size refinement. The experiment was performed by using two types of balls, 5 and 10 mm diameter, and exploring milling times up to 210 min. In addition to the particle size reduction, we observed changes in the morphology, structure and magnetic properties of the hydrogenated refined powders that were highly influenced by these two milling parameters. Deeper characterizations were performed for specific milling times in which significant changes to the magnetic properties were observed. The powders obtained using the 5 mm and 10 mm diameter balls were mainly investigated considering milling times of 5 to 30 min and 120 to 210 min, respectively.

**Figure 6:**
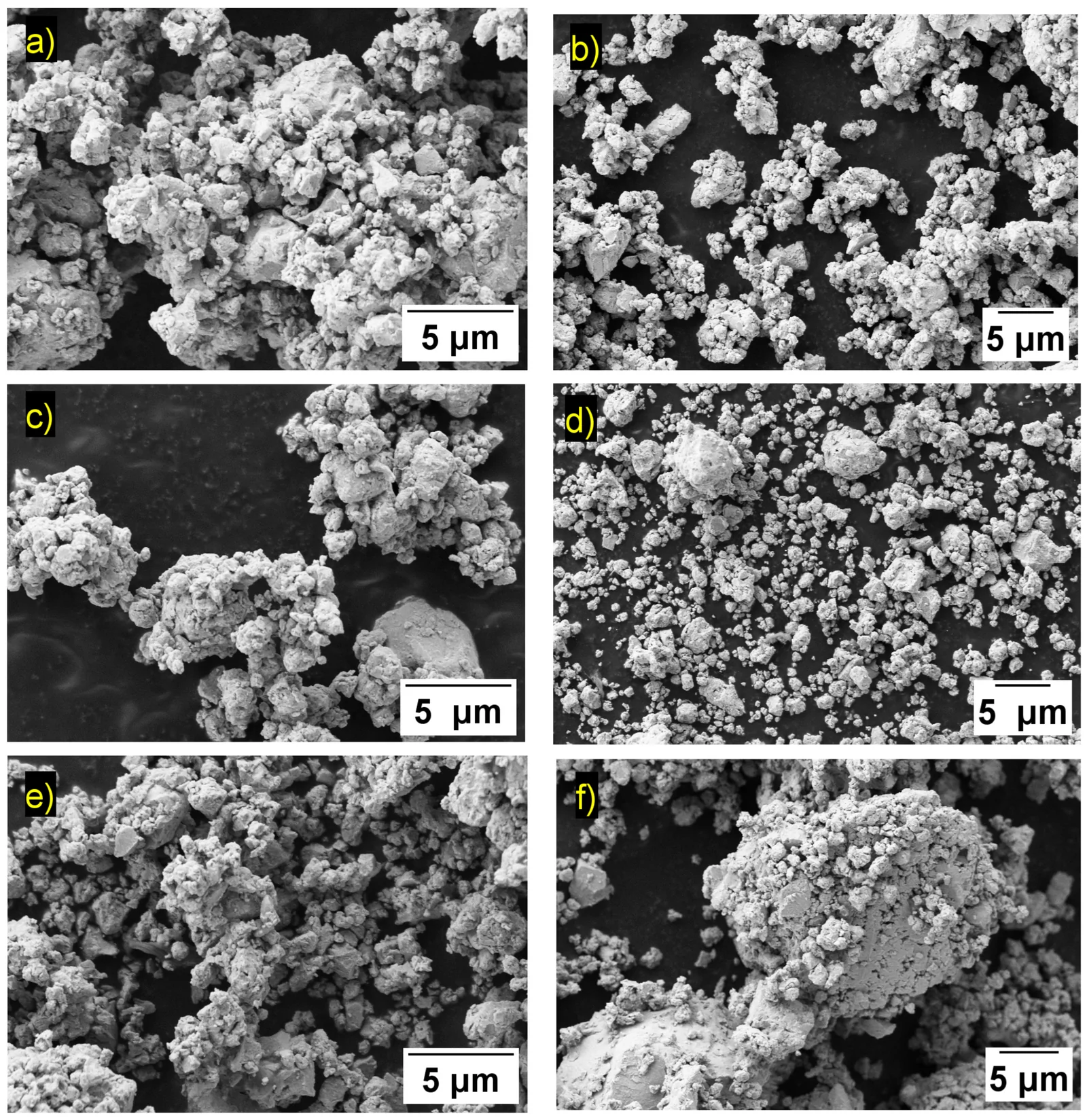
SEM images of the hydrogenated powders milled for 10 (**a**,**c**,**e**) and 30 (**b**,**d**,**f**) minutes using 5 mm balls from the processing of the three types of EoL magnets. Images (**a**,**b**) correspond to M1_68, (**c**,**d**) correspond to RMI, and (**e**,**f**) correspond to S21.

Regarding the ball-mill processing that employed 5 mm balls, Figure 6 displays SEM images of the hydrogenated powders of all the magnets milled for 10 and 30 min. For 10 min of milling, we observed powder fragmentation-formed particles of non-uniform size but round shapes, with particle sizes ranging from several hundred nanometers to a few μm. The particle size histograms reported in Figure S2 and Table 4 show that the average D_50_ is approximately 1.5 μm in all the powders, with a standard deviation between 1 and 3. After 30 min of milling, the particle size decreases further while aggregation is also observed. For longer milling times up to 150 min, (a mixture of fine particles between 200 nm and 2 μm was obtained.

**Figure 7:**
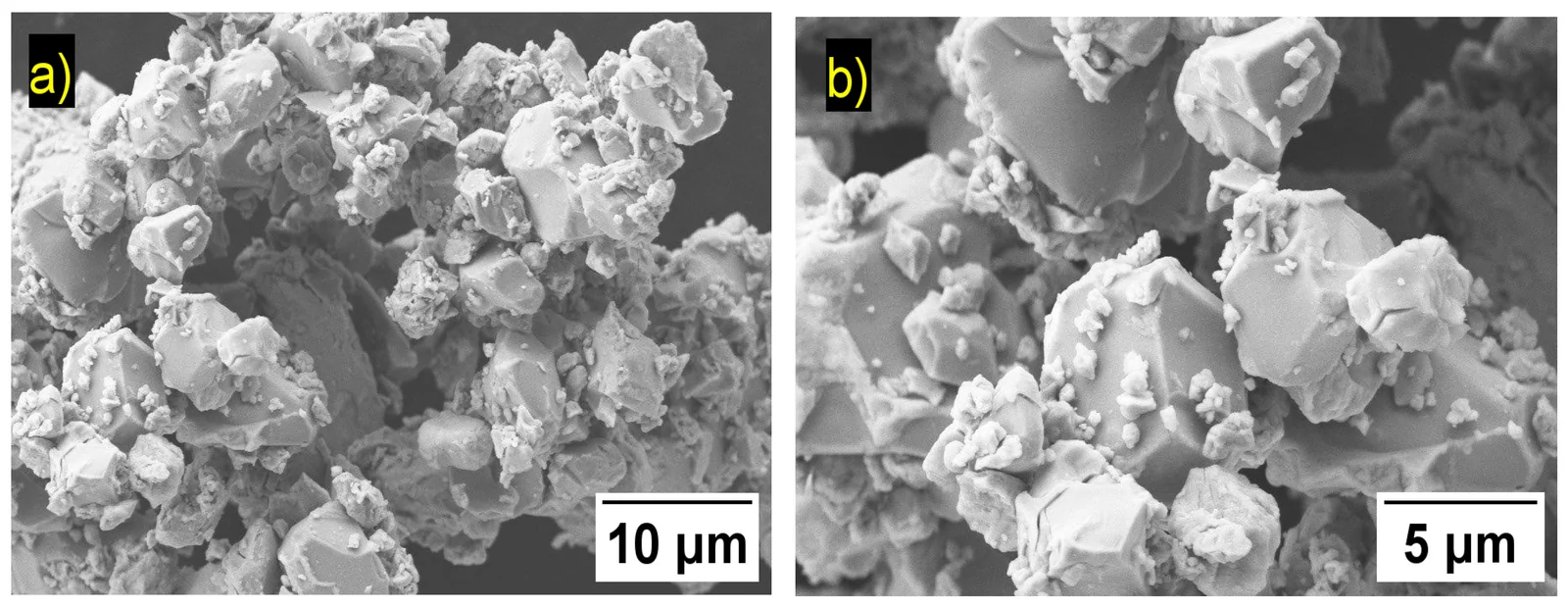
SEM images of hydrogenated M1_68 powders milled for 20 min using 10 mm balls: (**a**) 10 µm scale bar and (**b**) 5 µm scale bar.

**Figure 8:**
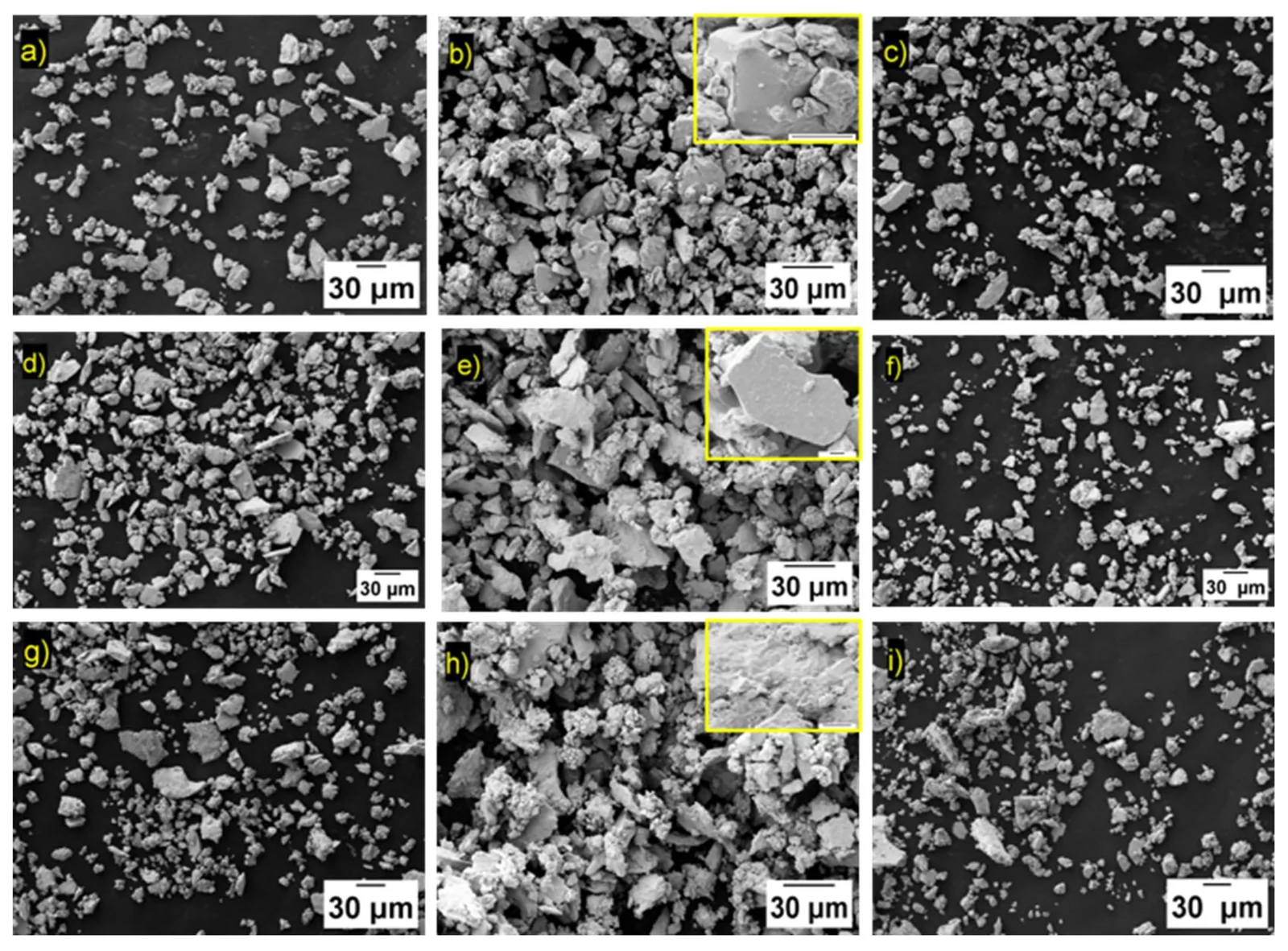
SEM images of hydrogenated powders milled for 120 (**a**,**d**,**g**), 150 (**b**,**e**,**h**) and 180 (**c**,**f**,**i**) minutes using 10 mm balls, respectively. Images (**a**–**c**) correspond to M1_68, (**d**–**f**) correspond to RMI and (**g**–**i**) correspond to S21. Insets show images acquired at greater magnification (3 µm).

The ball-mill processing employing 10 mm balls gives rise to powders with a different morphology. Figure 7 shows the SEM images of M1_68 powders milled for 20 min as representative of all the other powders. The original powders milled in this short time are fragmented to micron size of the original crystalline grains, retaining their polyhedral morphology. Also, submicrometric particles are observed. The particle size reduction and morphology changes are small compared to powders milled with 5 mm balls. For longer milling times, a complete change in morphology is observed. SEM images of the powders milled for 120, 150 and 180 min, shown in Figure 8, indicate that longer milling time leads to a loss of the original polyhedral morphology of the grains and the presence of agglomerates. Particle size distribution ranges from a few to tenths of microns with a progressive broadening as milling time increases. The smaller particles tend to form agglomerates or aggregates. In addition, as can be seen in the inset, fractured flake-like particles emerge, possibly due to brittle fractures of larger particles [43] caused by the intense impact of the balls, contributing to the uneven shape of the powders.

In all the cases of milling conditions, we observed similar morphologies for the three families of the powders. This implies that despite major differences in composition and recycling sources, ball milling produces similar morphological changes in the powders.

**Figure 9:**
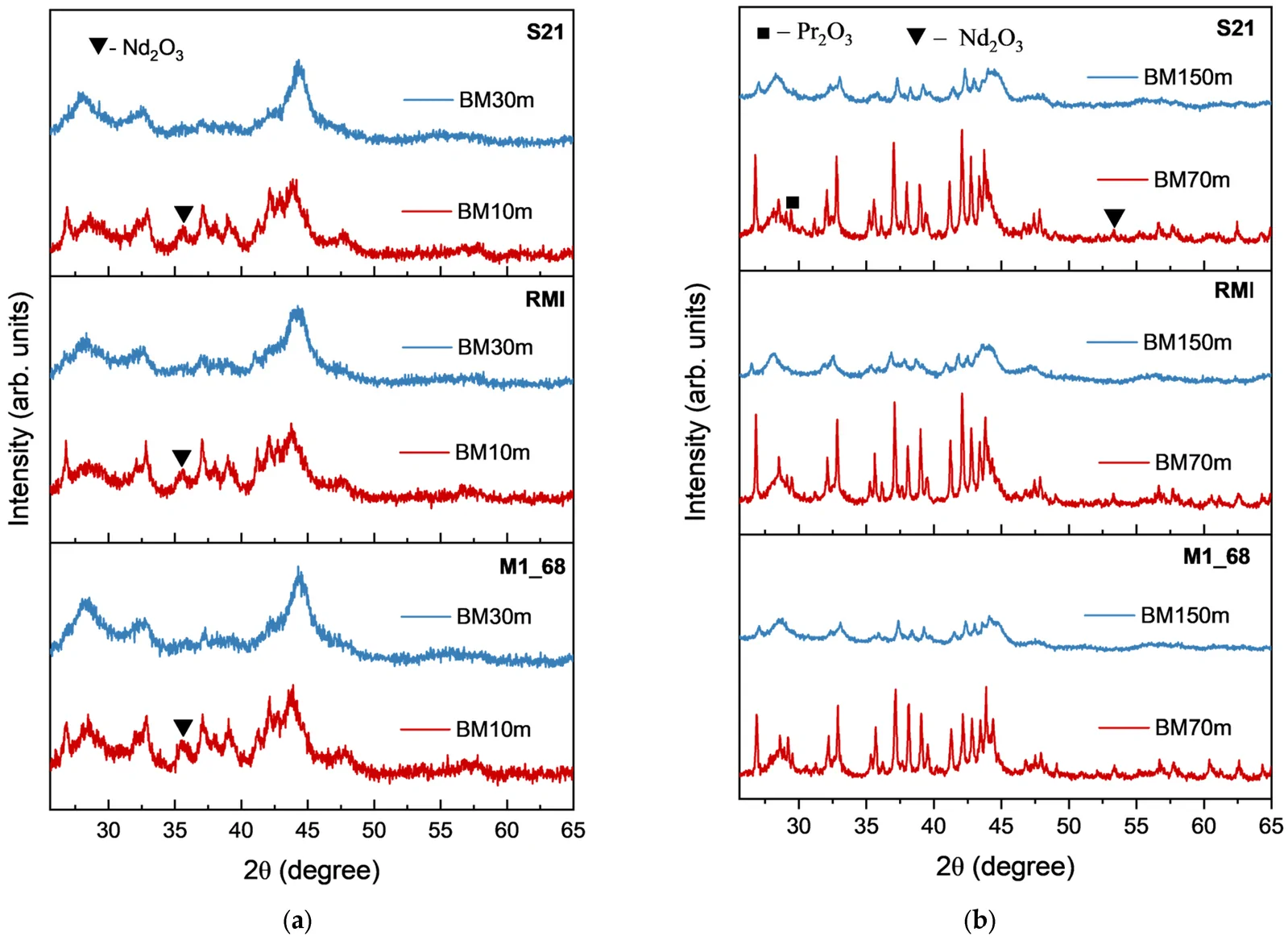
XRD patterns of (**a**) powders milled for 10 and 30 min using 5 mm balls; (**b**) powders milled for 70 and 150 min using 10 mm balls.

Using 5 mm balls for milling, the XRD patterns in Figure 9a show that the structural integrity of the particles changes rapidly with the milling time. After 10 min milling, a general broadening of all peaks is observed. After 30 min milling, only broad peaks are present. This broadening indicates crystalline grain size reduction but mainly structural degradation [44] arising from the milling process. During 10 min of milling, the (412) and (420) peaks that correspond to the hydrogenated P4_2_/mnm phase are present. After 30 min of milling, the dominant intensity peak around 44.5° is observed. This result indicates that the crystal structure of the hydrogenated powders changes because of the ball milling. In fact, the peak appears close to the diffraction angle of the (110) peak of α-Fe. Even if none of the other α-Fe reflections are present in the spectra, we cannot exclude the small segregation of this metal. However, from the XRD data we cannot obtain information to know the possible changes in the H content of the powders.

The XRD pattern of the ball-milled powders employing 10 mm diameter balls are represented in Figure 9b and show the structural evolution of the powders for each EoL magnet type. Within the limits of our experimental resolution, the peak positions do not change with respect to the coarse phase after 70 min of milling, suggesting that the milling process induces no modification of the hydrogen content. Also, we see that the peaks become broader. This change in the patterns suggests that damage to the crystal structure also increases when larger balls are used. The peaks corresponding to Pr_2_O_3_ and Nd_2_O_3_ are present for 70 min of milling. The peaks disappear for longer milling times, possibly due to amorphization.

Considering the previous results, we infer that the particle size of the balls influence strongly the milling processing, giving rise to materials with different microstructure and crystal structure. During milling with smaller balls, the particle size decreases faster but induces strong lattice defects [45]. For the milling with larger balls, the powders sustain less fragmentation and damage, possibly due to the smaller number of impacts and larger impact surfaces [46], but large milling times give rise to the formation of blended agglomerates of the fragmented powders. For both milling cases, the oxides are observed for shorter milling times but not for longer times. This could be due to their fragmentation and amorphization by the milling, and the oxide submicrometric particles are part of the agglomerates. This description covers the morphological and structural properties of the milled powders from the three types of magnets. This indicates that original compositions, structure and morphology do not give rise to different materials under mechanical processing.

**Figure 10:**
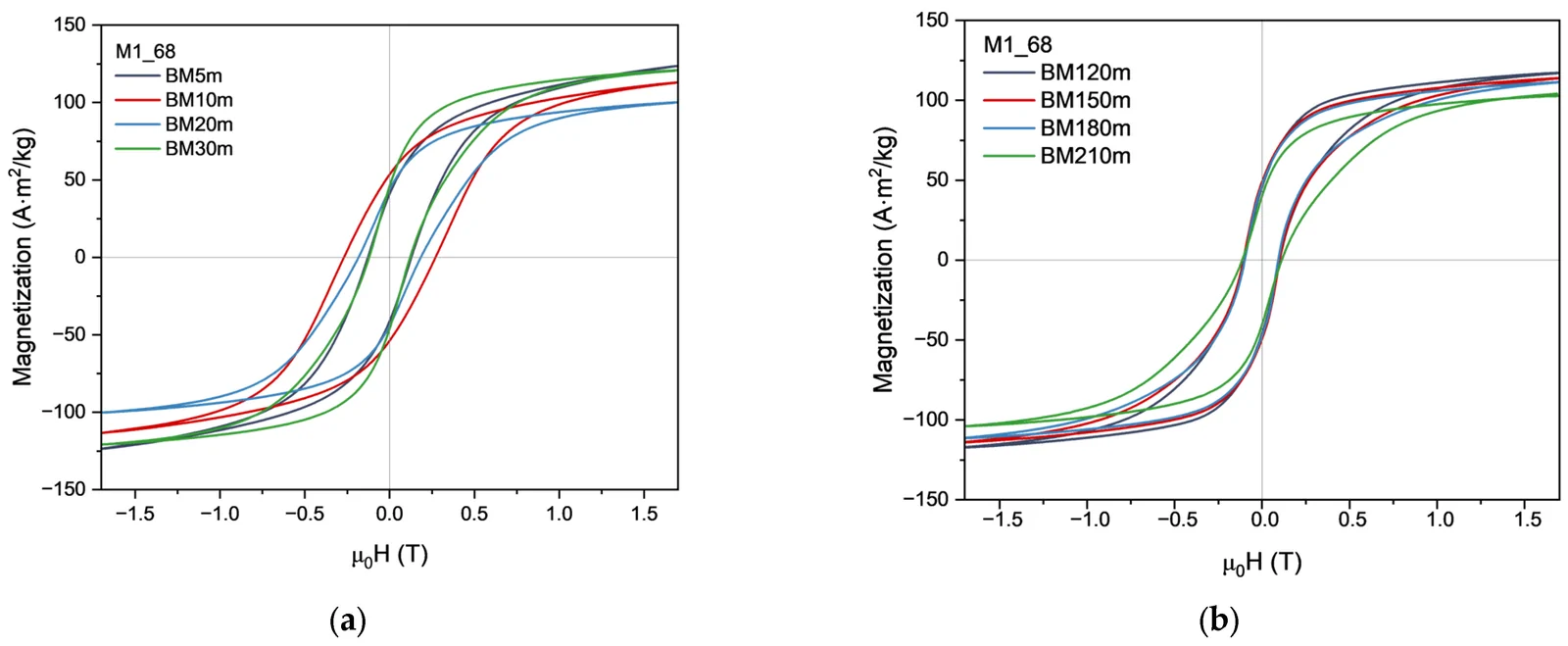
Hysteresis loops of M1_68 powders (**a**) milled for 5, 10, 20 and 30 min with 5 mm balls and (**b**) milled for 70, 120, 150 and 210 min with 10 mm balls.

The changes in the magnetic properties of the powders due to the microstructural refinement were investigated. Figure 10a and Figure 10b present the hysteresis loops of the M1_68 powders at different milling times and using 5 mm and 10 mm balls, respectively. With both types of balls, the coercive field and the saturation magnetization do not follow monotonic behavior as milling time increases. The specific magnetization decreases from 147 A·m^2^/kg of the coarse powder to values between 120 and 100 A·m^2^/kg, depending on the ball type and milling time. The specific magnetization decreases with the milling time, faster when employing the 5 mm balls than 10 mm balls. This effect is correlated to the magnetic disorder resulting from the structural damage induced by milling. This trend changes in the milling process with 5 mm balls during 30 min. The magnetization of these powders increases, reaching 125 A·m^2^/kg. This could be due to possible segregation of α-Fe, as previously discussed.

**Figure 11:**
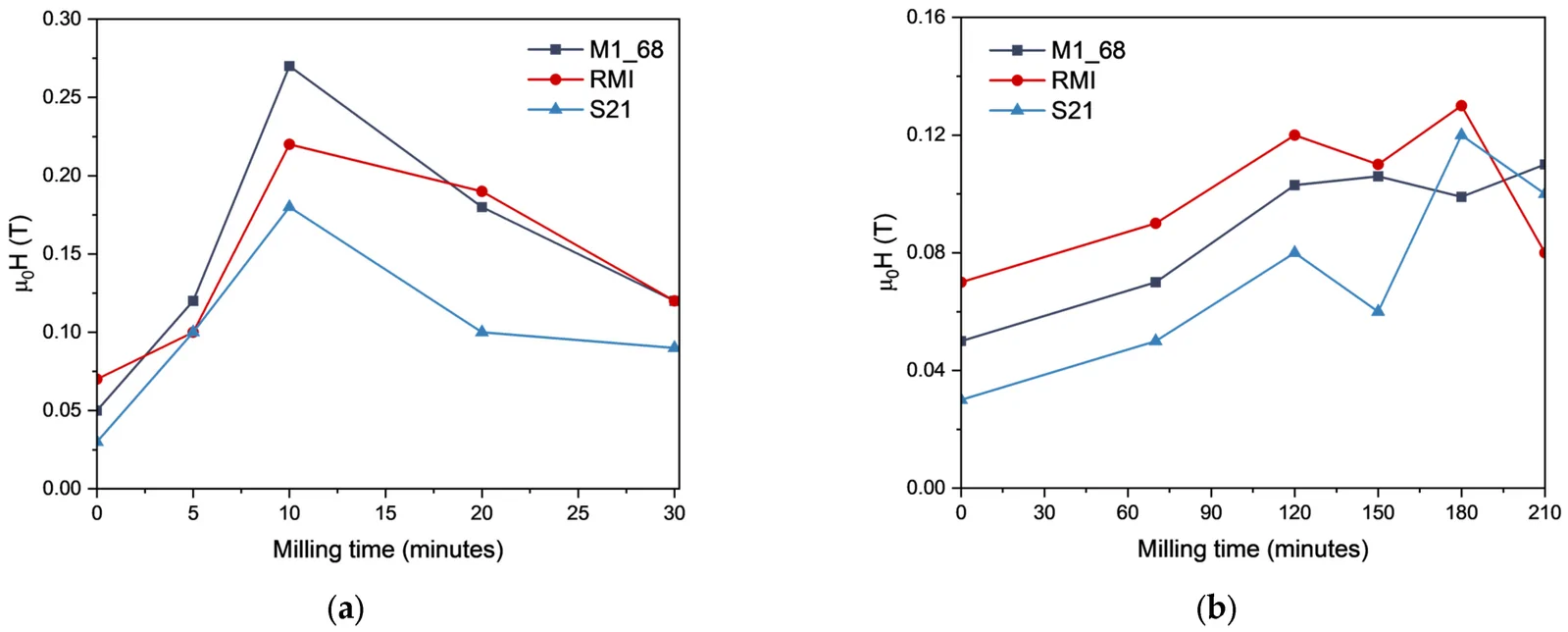
Coercive field as a function of milling time for M1_68, RMI and S21 powders: (**a**) milled with 5 mm balls (**b**) milled with 10 mm balls.

Regarding to the hysteresis loops, compared to the coarse HD powders, the ball-milled powders exhibit open loops that indicate the development of a microstructure in which magnetic irreversible processes occur. Employing the 5 mm balls, the Hc of the powders from all three magnets reaches a maximum after 10 min milling, as shown in Figure 11a. In fact, Hc increases 2.5-, 4.5- and 5-fold for RMI, M1_68 and S21, respectively, from the corresponding hydrogenated powders. The coercive field of the powders milled with 10 mm balls increases slowly to reach a maximum that differs from magnet to magnet, as shown in Figure 11b. Hc increases less than in the previous case: 1-, 1.2- and 3-fold for RMI, M1_68 and S21, respectively, from the initial powders to the milled ones. In all cases, S21 powders exhibit the largest increase in Hc. The powders from the M1_68 magnets obtained after milling 10 min with the 5 mm balls present the larger value of Hc, 0.27 T, with respect to the others. When employing the 5 mm and 10 mm balls, further milling for more than 30 min and 210 min, respectively, causes a decline in the coercivity.

**Figure 12:**
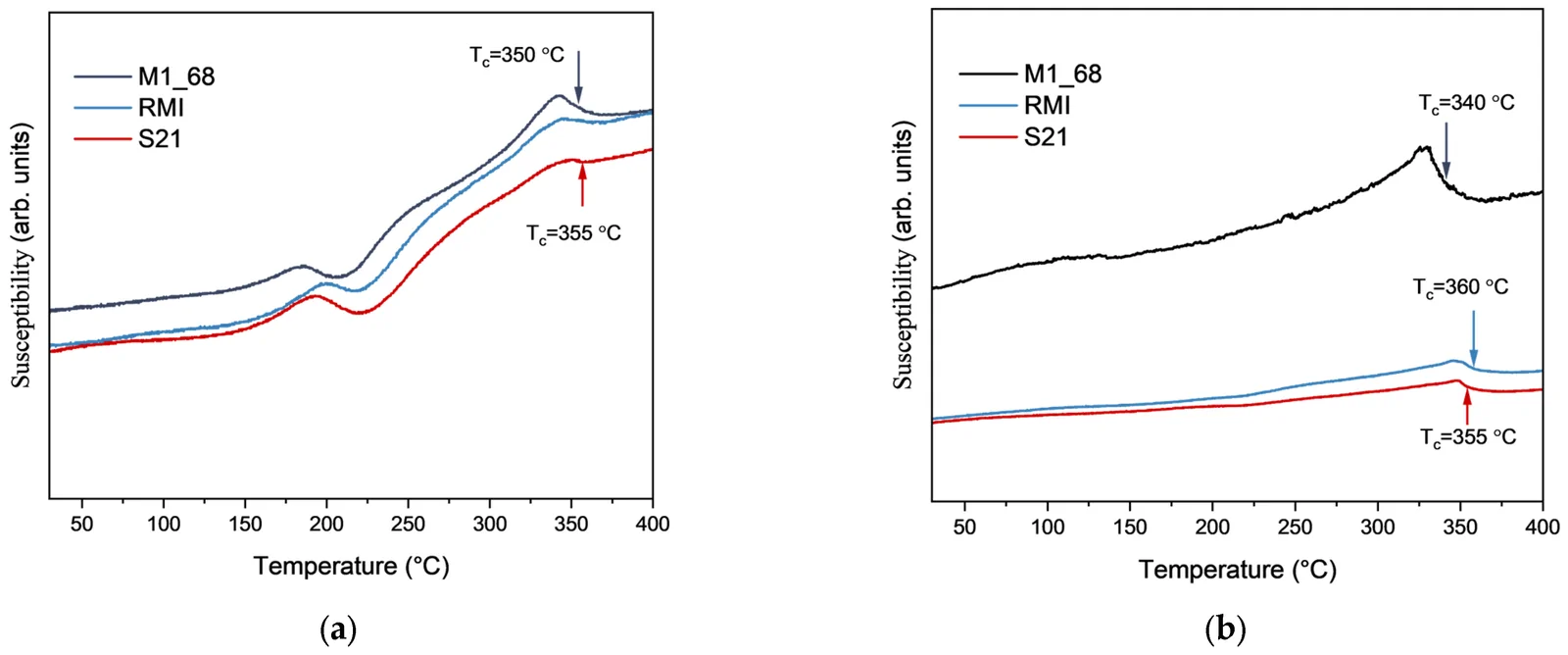
Thermomagnetic measurement of powders (**a**) milled for 10 min with 5 mm balls and (**b**) milled for 150 min with 10 mm balls.

The magnetic properties of the ball-milled powders with the highest coercive fields were investigated by thermomagnetic analysis. The susceptibility curves as a function of temperature, presented in Figure 12a,b, demonstrate the presence of several magnetic phases. In the case of the samples milled with 5 mm balls, two peaks near 200 °C and 360 °C are present, which can be correlated with the presence of two phases. In addition, the susceptibility signal does not reach zero at Tc = 360 °C, indicating the presence of a third magnetic phase with a higher Tc than the hydrogenated phase. This could be α-Fe (Tc = 711 °C) or FeB phases (550 °C for FeB and around 740 °C for Fe_2_B) [47]. Considering the XRD structural studies, the peaks of the hydrogenated Nd_2_Fe_14_B patterns are broad and shifted with respect to the known phases, and hence it is not possible to precisely identify all the magnetically ordered phases detected by thermomagnetic measurements.

The Tc values of the three types of powders milled with 5 mm balls fall within the same temperature range (between 355 °C and 360 °C), as reported in Table 8. If this phase is associated with the hydrogenated phase detected by XRD, our results indicate that its composition is relatively homogeneous in all three cases, with an increase in T_C_ compared with that of the original hydrogenated powders. The XRD studies show that the crystal structure of the hydrogenated NdFeB phase is strongly defective in all cases. Our results suggest that the resulting effect is an unexpected increase in Tc and the homogenization of Tc independent of the differences in REE composition. The decrease in Nd content was correlated with the observed oxidation; alternatively, H desorption could play a role.

The powders milled with 10 mm balls exhibit different magnetic properties compared to those milled with 5 mm balls. The thermomagnetic measurements, provided in Figure 12b, show smaller changes in the susceptibility in all the samples, with the peaks corresponding to values between 340 and 352 °C, and a phase with larger Tc as well. As reported in Table 8, the Tc values are similar to those of the hydrogenated powders, suggesting that the milling process with large balls gives rise to very small elemental composition changes in comparison to the effects induced by the use of small balls.

A key result of our study is that the coercive field increases in a certain milling time, reaching a maximum value that is at short 10 min times for the small 5 mm balls and long 180 min times for large balls. The coercive field depends on the intrinsic magnetic anisotropy of the material, but also on the extrinsic properties like the morphology, crystal structure and presence of secondary phases, density, and composition. As discussed previously, the magnetic anisotropy of the hydrides could not be considered negligible, with anisotropy fields reaching values up to 3 T [36]. The increase in H_C_ could be related to the substantial reduction in particle size of the hydrogenated powders to the micrometric scale, where the probability of domain-wall nucleation may decrease as the particle size approaches the single-domain size [48]. However, this effect should have a limit in the co-existence of agglomeration processes, as it is observed in the powders milled with 10 mm diameter balls, as stronger bipolar interparticle interactions limit the beneficial effect of the size reduction in the coercive field. Also, we have shown the progressive damage of the crystallinity that increases with the milling time. This produces the decrease in the magnetic order and atomic magnetic anisotropy [49].

We propose a second possible mechanism that could give rise to the observed increase in H_C_ due to the milling. Our hypothesis is that the energy transfer by the ball collision could induce not only particle fragmentation and material damage, but also a local increase in temperature, which is sufficient to induce local desorption of hydrogen. This will create the presence of local partially dehydrogenated 2:14:1 nanostructures with higher anisotropy that induces the average increase in the effective anisotropy and the coercive field of the powders. The effect can also be described as a local degassing of the hydrogenated NdFeB and growth of the 2:14:1 phase. In fact, HPMSs include a high-temperature degassing step that directly produces the final hard phase [50]. In our case, we observe that the milling with 5 mm balls is more energetic, producing a more efficient local degassing and growth of hard nanophase, which could explain the larger H_C_. Considering that the structural and morphological properties of the processed powders from different magnets seem similar, we propose that the variation in the coercive field could be correlated with the different content of REEs present in the magnets that influence the effective magnetic anisotropy. This is the first factor that appears different between the powders derived from different magnets. On the other hand, this improvement is contrasted by the structural damage and/or the particle agglomeration that occurs with the milling time, giving rise to the final observed decrease in the coercive field. Finally, we point out that the observed H_C_ variations with the milling time and balls are very similar in the powders from the three types of EoL magnets. This suggests that the elemental composition of the EoL magnets does not play an important role in the morphological and structural transformations of the hydrogenated powders during milling.

## 4. Conclusions

Our fundamental and laboratory-scale study demonstrates the possibility of particle refining of hydrogenated Nd-Fe-B-based powders using high-energy ball-milling processing for the final scope of recycling end-of-life magnets. The magnets were decrepitated under hydrogen at room temperature, and the hydrogenated powders were processed using different ball sizes and milling times, which controlled the microstructure. The micron to submicrometer powders can be obtained in short milling times, 10–20 min, by using 5 mm size balls. When using the larger 10 mm balls, longer milling times were required to reduce the particle size of the powders. In this case, the particle size ranges from a few microns to tenths of microns, and the powders are characterized by irregular shapes and fractured flake-like particles. In both milling cases, particles form agglomerates. In addition, a strong structural disorder is observed, mainly when using the smaller balls.

Nd and Nd/Pr oxides are observed in the pristine powders and initially during milling, but for longer milling times they are undetected by XRD, possibly due to amorphization. The presence of oxygen is a critical issue for sintered magnet production. Thus, in this case, detection requires precise and localized analytical techniques.

The magnetic properties are also modified by the milling process and depend on the ball choice. The magnetization saturation decreases with the milling time, indicating a loss of magnetic order. Contrarily, the coercive field exhibits non-monotonic behavior characterized by a peak in the coercive field at different milling time scales depending on the ball size. The highest coercive field value of 0.27 T was obtained in the hydrogenated powders from a wind turbine magnet after 10 min milling using 5 mm balls. The relatively high coercive fields of hydrogenated powders have not previously been reported in the literature. The evolution of Hc is interpreted by considering competition between several structural and compositional effects: the particle size reduction and the possible milling-induced growth of a partial de-hydrogenated hard phase that gives rise to Hc, while milling-induced structural damages and particle agglomeration produces the opposite effect. Monitoring the Curie temperature of the milled powders confirms compositional or structural changes in the hydrogenated powders induced by the milling process and indicate the segregation of a secondary phase, mainly evident in the powders milled with small balls.

Our study also examines the recycling process for different types of EoL magnets. For the moment, most of the recycling technologies preview the transformation/processing of magnets from different origins. Our results provide insights into how the properties of the EoL magnets are transferred to their respective hydrides and milled powders, as a step in the magnet-to-magnet recycling process. This is particularly important as the most likely approach to magnet production involves processing mixtures of various types of EoL magnets. To observe the differences in the powders and properties, we monitored the milling process in three types of magnets that exhibit different composition and magnetic properties. The initial composition of the magnets, mainly the rare earth content, was shown to influence the coercive field and Curie temperature of both the hydrides and milled powders, but the morphology and structural properties do not seem to be affected. This study helps to understand the complex microstructural evolution during the recycling process of EoL NdFeB magnets into recycled powders from different sources that come with varying compositions. This research focused on the HPMSs step, which is part of the magnet-to-magnet recycling process. Future studies will investigate the influence of different morphologies on the degassing process and subsequent hard-phase formation during the production of recycled magnets.

This study shows new physicochemical phenomena that may arise during particle refinement process, not only for recycling magnets (e.g., HPMSs, which employs jet milling for refinement), but also in the processing of other functional or multi-functional magnetic materials.

## Figures and Tables

**Table 1:** The elemental composition of the three types of magnets after hydrogenation.

Source	Name	Composition (%wt)														
		Nd	Al	Co	Cu	Gd	Dy	Ho	Nb	Pr	Tb	P	Si	Ce	Fe	B
Wind turbine	M1_68	33.58	0.49		0.22		3.27			0.85		0.38	0.36		56.21	0.88
Ring magnet	RMI	21.76	0.58	1.09	0.17		3.02	1.13	0.11	4.53	0.53	0.37	0.33		64.70	1.04
Scooter	S21	20.96	0.93	0.82	0.16	3.06		0.26	0.01	5.77		0.39	0.31	1.60	64.70	1.03

**Table 2:** Calculated lattice parameters, cell volumes, weighted profile residual (Rwp), and profile residual factors (Rp) of the Rietveld analysis for EoL magnets.

Sample		EoL Magnets			
	a (Å)	c (Å)	V (Å^3^)	Rwp (%)	Rp (%)
M1_68	8.791	12.182	815.2	6.8	5.0
RMI	8.791	12.174	814.6	6.2	4.9
S21	8.789	12.203	816.1	6.5	5.0

**Table 3:** Summary of the magnetic properties of the EoL magnets.

Sample	Magnetization (A·m^2^/kg)	Coercive Field (T)	Curie Temperature (°C)
M1_68	141	1.8	310
RMI	147	1.9	337
S21	137	1.85	317

**Table 5:** Calculated lattice parameters, cell volumes, weighted profile residual (Rwp), and profile residual factors (Rp) of the Rietveld analysis for the HD powders.

Sample		HD			
	a (Å)	c (Å)	V (Å^3^)	Rwp (%)	Rp (%)
M1_68	8.840	12.249	828.9	1.57	0.94
RMI	8.878	12.297	839.5	1.70	1.01
S21	8.870	12.311	838.7	2.70	1.48

**Table 6:** Calculated percentage of lattice parameter and volume expansions of the powders after hydrogen decrepitation.

Sample	Δa (%)	Δc (%)	ΔV (%)
M1_68	0.55	0.56	1.69
RMI	0.98	1.02	2.48
S21	0.92	0.89	2.43

**Table 7:** Summary of magnetic properties of the HD powders.

Sample	Saturation Magnetization (A·m^2^/kg)	Coercive Field (mT)	Curie Temperature (°C)
M1_68_HD	148	50	340
RMI_HD	145	40	350
S21_HD	143	30	350

**Table 8:** Curie temperature of ball-milled powders with 5 mm balls for 10 min and with 10 mm balls for 150 min.

Sample	Curie Temperature (°C)	
	5 mm Balls	10 mm Balls
M1_68	360	340
RMI	358	352
S21	355	350

## Data Availability

The original contributions presented in this study are included in the article/Supplementary Materials. Further inquiries can be directed to the corresponding author.

## Supplementary Materials

The following supporting information can be downloaded at: https://www.mdpi.com/article/10.3390/ma19173797/s1. Figure S1: XRD patterns and corresponding theoretical Rietveld profiles of the RMI magnet (RMI_magnet) and RMI hydrogenated (RMI_HD) powders. Figure S2: Particle size histograms and corresponding lognormal distribution of the HD powders from the different EoL magnets and those milled for 20 min using 5 mm balls and milled for 150 min using the 10 mm balls. The histograms were obtained from analysis of SEM images at high magnification.

## References

[1] Sagawa M., Fujimura S., Togawa N., Yamamoto H., Matsuura Y. (1984). New material for permanent magnets on a base of Nd and Fe (invited). J. Appl. Phys..

[2] Woodcock T.G., Zhang Y., Hrkac G., Ciuta G., Dempsey N., Schrefl T., Gutfleisch O., Givord D. (2012). Understanding the microstructure and coercivity of high performance NdFeB-based magnets. Scr. Mater..

[3] Gauß R., Burkhardt C., Carencotte F., Gasparon M., Gutfleisch O., Higgins I., Karajić M., Klossek A., Mäkinen M., Schäfer B. (2021). Rare Earth Magnets and Motors: A European Call for Action. https://erma.eu/european-call-for-action/.

[4] Ghorbani Y., Ilankoon I.M.S.K., Dushyantha N., Nwaila G.T. (2025). Rare earth permanent magnets for the green energy transition: Bottlenecks, current developments and cleaner production solutions. Resour. Conserv. Recycl..

[5] Gutfleisch O., Willard M.A., Brück E., Chen C.H., Sankar S.G., Liu J.P. (2011). Magnetic Materials and Devices for the 21st Century: Stronger, Lighter, and More Energy Efficient. Adv. Mater..

[6] Lixandru A., Poenaru I., Güth K., Gauß R., Gutfleisch O. (2017). A systematic study of HDDR processing conditions for the recycling of end-of-life Nd-Fe-B magnets. J. Alloys Compd..

[7] Binnemans K., Jones P.T., Blanpain B., Van Gerven T., Yang Y., Walton A., Buchert M. (2013). Recycling of rare earths: A critical review. J. Clean. Prod..

[8] Zakotnik M., Harris I.R., Williams A.J. (2008). Possible methods of recycling NdFeB-type sintered magnets using the HD/degassing process. J. Alloys Compd..

[9] Mo C.C., Kuan C.-C., Wang Y.-H., Lu Y.-S., Chang T.-W., Liao W.-Y., Fang T.-H., Tsai M.-C., Huang C.-C. (2023). Effect of NdCoGa alloy addition to waste wind turbine magnets to enhance the characteristics of recycled sintered NdFeB permanent magnets. J. Magn. Magn. Mater..

[10] Prosperi D., Bevan A., Ugalde G., Tudor C., Furlan G., Dove S., Lucia P., Zakotnik M. (2018). Performance comparison of motors fitted with magnet-to-magnet recycled or conventionally manufactured sintered NdFeB. J. Magn. Magn. Mater..

[11] Li X., Yue M., Zakotnik M., Liu W., Zhang D., Zuo T. (2015). Regeneration of waste sintered Nd-Fe-B magnets to fabricate anisotropic bonded magnets. J. Rare Earths.

[12] Kumari A., Sahu S.K. (2023). A comprehensive review on recycling of critical raw materials from spent neodymium iron boron (NdFeB) magnet. Sep. Purif. Technol..

[13] Deng C., Zhang X., Guo F., Wang S., Wang X. (2025). The current status of recycling technology for waste NdFeB resources. J. Mater. Cycles Waste Manag..

[14] Nayebossadri S., Awais M., Arnold R., Degri M., Mann N., Walton A. (2024). Hydrogen-assisted recycling of Nd-Fe-B magnets from the end-of-life audio products. J. Magn. Magn. Mater..

[15] Li C., Yue M., Liu W., Zuo T., Yi X., Chen J., Zhou Z., Wu Y. (2015). Recycling of scrap sintered Nd–Fe–B magnets as anisotropic bonded magnets via hydrogen decrepitation process. J. Mater. Cycles Waste Manag..

[16] Poenaru I., Patroi E.A., Patroi D., Iorga A., Manta E. (2023). HDDR as advanced processing method and recycling technology to address the rare-earth resource criticality in high performance Nd2Fe14B magnets production. J. Magn. Magn. Mater..

[17] Harris I.R., McGuiness P.J. (1991). Hydrogen: Its use in the processing of NdFeB-type magnets. J. Less Common Met..

[18] Ragg O.M., Keegan G., Nagel H., Harris I.R. (1997). The HD and HDDR processes in the production of Nd-Fe-B permanent magnets. Int. J. Hydrogen Energy.

[19] Zakotnik M., Devlin E., Harris I.R., Williams A.J. (2006). Hydrogen Decrepitation and Recycling of NdFeB-type Sintered Magnets. J. Iron Steel Res. Int..

[20] Burkhardt F., Berja A., Grau L., Kreča M., Krasniqi L., Podmiljšak B., Žužek K., Burkhardt C., Kobe S., Quesada A. (2025). A Rapid Consolidation Route for Recycled NdFeB Powders and the Role of Particle Shape in Grain Growth. Materials.

[21] Grau L., Burkhardt F., Moll N., Rathfelder S., Kobe S., Burkhardt C. (2025). Effects of Contamination on the Recyclability of NdFeB Permanent Magnets via Short-Loop Processing: Review of Common Contaminants and Study on Ni Coating Residues. Recycling.

[22] Mishra A., Khoshsima S., Tomše T., Podmiljšak B., Šturm S., Burkhardt C., Žužek K. (2023). Short-Loop Recycling of Nd-Fe-B Permanent Magnets: A Sustainable Solution for the RE2Fe14B Matrix Phase Recovery. Materials.

[23] Diehl O., Schönfeldt M., Brouwer E., Dirks A., Rachut K., Gassmann J., Güth K., Buckow A., Gauß R., Stauber R. (2018). Towards an Alloy Recycling of Nd–Fe–B Permanent Magnets in a Circular Economy. J. Sustain. Metall..

[24] Nakamura M., Matsuura M., Tezuka N., Sugimoto S., Une Y., Kubo H., Sagawa M. (2013). Preparation of ultrafine jet-milled powders for Nd-Fe-B sintered magnets using hydrogenation-disproportionation-desorption-recombination and hydrogen decrepitation processes. Appl. Phys. Lett..

[25] McGuiness P.J., Devlin E., Harris I.R., Rozendaal E., Ormerod J. (1989). A study of Nd-Fe-B magnets produced using a combination of hydrogen decrepitation and jet milling. J. Mater. Sci..

[26] Xia M., Abrahamsen A.B., Bahl C.R.H., Veluri B., Søegaard A.I., Bøjsøe P. (2017). Hydrogen Decrepitation Press-Less Process recycling of NdFeB sintered magnets. J. Magn. Magn. Mater..

[27] Degen T., Sadki M., Bron E., König U., Nénert G. (2014). The HighScore suite. Powder Diffr..

[28] Grohol M., Veeh C., European Commission, Directorate-General for Internal Market, Industry, Entrepreneurship and SMEs (2023). Study on the Critical Raw Materials for the EU 2023—Final Report.

[29] Geshev J., Popov O., Mikhov M., Ll J.L.S., Suarez N., Leccabue F. (1992). The Hopkinson effect in a BaFe12O19 fine particle system: Demagnetization field effects. J. Magn. Magn. Mater..

[30] Song X.M., Zhang T.A., Dou Z.H., Pei W.L., Zhou L. (2020). Effects of Dy diffusion time on magnetic properties of Nd-Fe-B sintered magnets. Mater. Res. Express.

[31] Baloyi M.E., Diale R.G., Ngoepe P.E., Chauke H.R. (2025). Effect of Terbium on the magnetic properties of Nd2Fe14B permanent magnets. MRS Adv..

[32] Liu Z.W., Davies H.A. (2006). Influence of Co substitution for Fe on the magnetic properties of nanocrystalline (Nd,Pr)-Fe-B based alloys. J. Phys. D Appl. Phys..

[33] Habibzadeh A., Kucuker M.A., Çakır Ö., Gökelma M. (2024). Microstructural Investigation of Discarded NdFeB Magnets After Low-Temperature Hydrogenation. J. Sustain. Metall..

[34] Nikitin S.A., Tereshina I.S., Pankratov N.Y., Palewski T., Drulis H., Makarova M.V., Pastushenkov Y.G. (2003). Effect of hydrogen on the magnetic characteristicsof Nd2Fe14B single crystal. Phys. Status Solidi (A).

[35] Andreev A.V., Deryagin A.V., Kudrevatykh N.V., Mushnikov N.V., Reimer V.A., Terent’ev S.V. (1986). Magnetic properties of Y2Fe14B and Nd2Fe14B and their hydrides. Sov. Phys. JETP.

[36] Mushnikov N.V., Terent’Ev P.B., Rosenfel’D E.V. (2007). Magnetic anisotropy of the Nd2Fe14B compound and its hydride Nd2Fe14BH4. Phys. Met. Metallogr..

[37] Li J., Sepehri-Amin H., Sasaki T., Ohkubo T., Hono K. (2021). Most frequently asked questions about the coercivity of Nd-Fe-B permanent magnets. Sci. Technol. Adv. Mater..

[38] Givord D., Rossignol M., Barthem V.M.T.S. (2003). The physics of coercivity. J. Magn. Magn. Mater..

[39] Sagawa M., Hirosawa S. (1988). Coercivity and microstructure OF R-Fe-B sintered permanent magnets. J. Phys. Colloq..

[40] Kronmüller H. (1987). Theory of Nucleation Fields in Inhomogeneous Ferromagnets. Phys. Status Solidi (B).

[41] Wiesinger G., Hilscher G. (2007). Chapter Five Magnetism of Hydrides. Handbook of Magnetic Materials.

[42] Pareti L., Moze O., Fruchart D., L’Heritier P., Yaouanc A. (1988). Effects of hydrogen absorption on the 3d and 4f anisotropies in RE2Fe14B (RE ≡ Y, Nd, Ho, Tm). J. Less Common Met..

[43] Shin D.W., Madavali B., Kim D.-S., Lee J.-Y., Kang M.-C., Yang C.-W., Suryanarayana C., Hong S.-J. (2020). Investigation of the magnetic properties and fracture behavior of Nd-Fe-B alloy powders during high-energy ball milling. Mater. Res. Express.

[44] Jurczyk M., Cook J.S., Collocott S.J. (1995). Application of high energy ball milling to the production of magnetic powders from NdFeB-type alloys. J. Alloys Compd..

[45] Miao W.F., Mccormick P., Street R., Ding J. (1996). Effect of Mechanical Milling on the Structure and Magnetic Properties of Nd16Fe76B8. J. Phys. D-Appl. Phys..

[46] Mural Z., Kollo L., Traksmaa R., Kallip K., Link J., Veinthal R. (2014). Structure and magnetic properties of NdFeB powder prepared by hydrogen decrepitation and high-energy ball milling. Key Engineering Materials.

[47] Wang D., Ma L., Li L., Xu X.L., Guo Y.B., Zhao S.Q. (2018). Characterization of Polycrystalline Fe2B Compound with High Saturation Magnetization. J. Supercond. Nov. Magn..

[48] Sugimoto S., Nakamura M., Matsuura M., Une Y., Kubo H., Sagawa M. (2015). Enhancement of Coercivity of Nd-Fe-B Ultrafine Powders Comparable with Single-Domain Size by the Grain Boundary Diffusion Process. IEEE Trans. Magn..

[49] Su K.P., Liu Z.W., Zeng D.C., Huo D.X., Li L.W., Zhang G.Q. (2013). Structure and size-dependent properties of NdFeB nanoparticles and textured nano-flakes prepared from nanocrystalline ribbons. J. Phys. D Appl. Phys..

[50] Book D., Harris I.R. (1995). Hydrogen absorption/desorption and HDDR studies on Nd16Fe76B8 and Nd11.8Fe82.3B5.9. J. Alloys Compd..

