# Supplementary material for: Ball-Milling Processing of Hydrogenated NdFeB Powders from the Recycling of End-of-Life Magnets
Source: Materials (Basel). 2026 Sep 6;19(17):3797. doi: 10.3390/ma19173797 (PMC13567385; doi:10.3390/ma19173797)
Supplement: Supplementary file 1 [file materials-19-03797-s001.zip › materials-4461487-supplementary.pdf]

Supplementary Information of article ''Ball Milling processing of hydrogenated NdFeB powders from the recycling of end-of-life magnets '' by Wako et al.

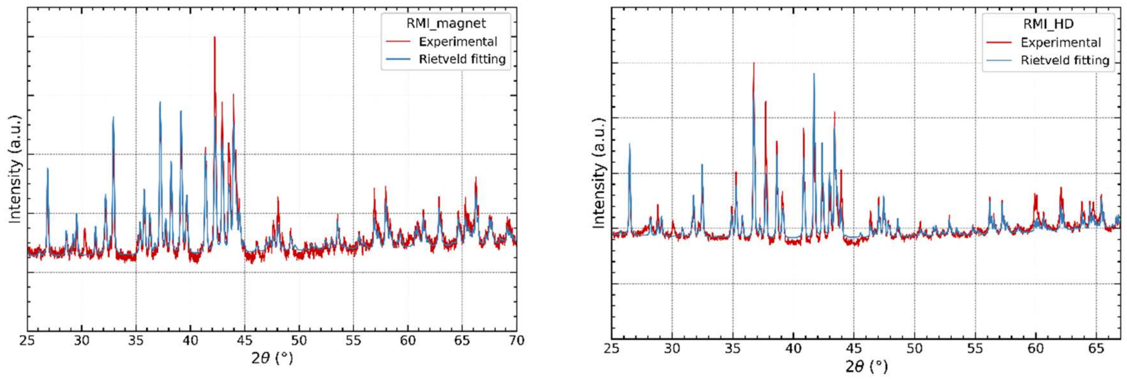

(**a**) (**b**)

Figure S1. XRD patterns and corresponding theoretical Rietveld profiles of the a) RMI magnet (RMI_magnet), and b) RMI hydrogenated (RMI_HD) powders.

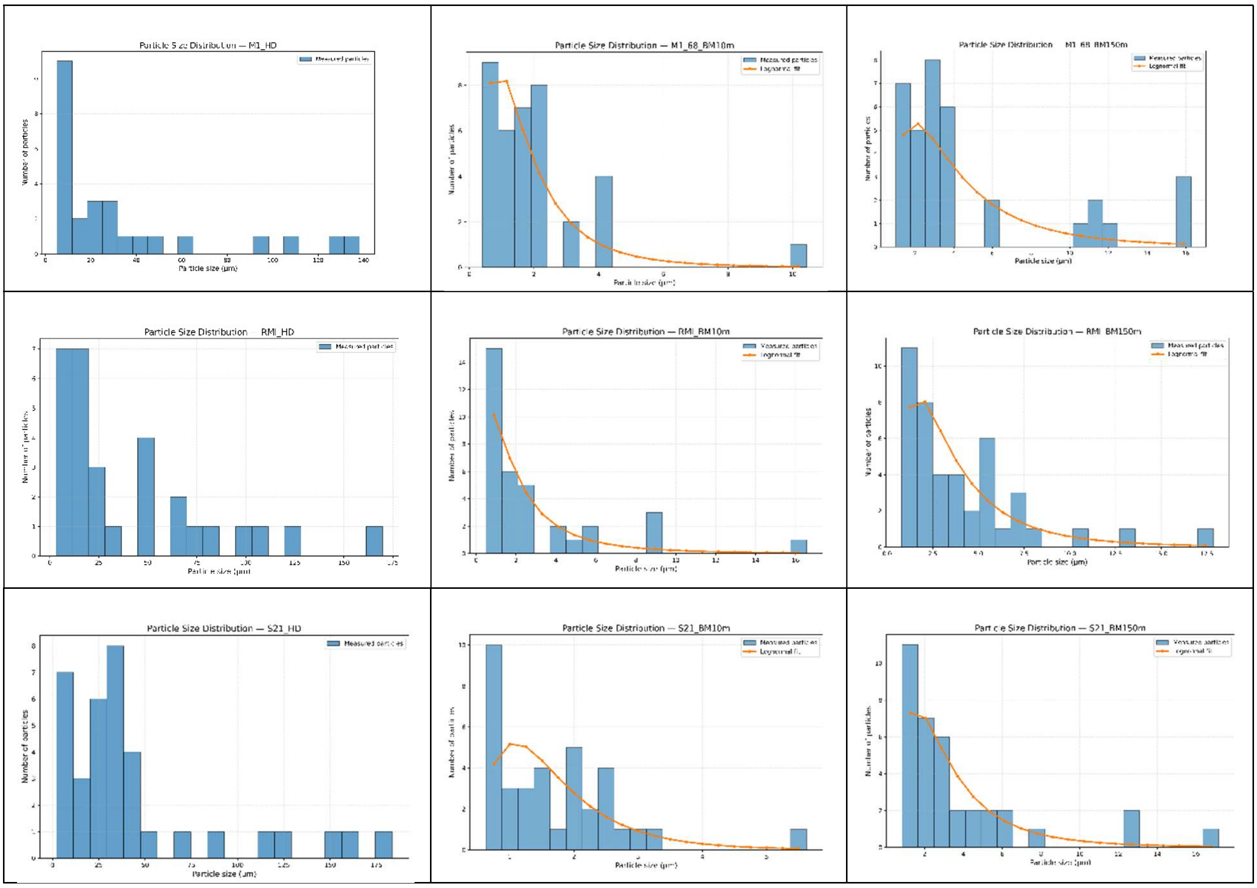

Figure S2. Particle size histograms and corresponding lognormal distribution of the HD powders from the different EoL magnets and those milled for 10 min using 5mm balls and milled for 150 min using the 10 mm balls. The histograms were obtained from analysis of SEM images at high magnification.
